# Volumetric Additive Manufacturing of Dicyclopentadiene by Solid‐State Photopolymerization

**DOI:** 10.1002/advs.202402385

**Published:** 2024-07-05

**Authors:** Matthew M. Hausladen, Esteban Baca, Kyle A. Nogales, Leah N. Appelhans, Bryan Kaehr, Craig M. Hamel, Samuel C. Leguizamon

**Affiliations:** ^1^ Chemical Engineering and Materials Science University of Minnesota Minneapolis MN 55455 USA; ^2^ Sandia National Laboratories Albuquerque NM 87185 USA

**Keywords:** additive manufacturing, photopolymerization, plastic crystal, ring‐opening metathesis polymerization, solid state, volumetric additive manufacturing

## Abstract

Polymerization in the solid state is generally infeasible due to restrictions on mobility. However, in this work, the solid‐state photopolymerization of crystalline dicyclopentadiene is demonstrated via photoinitiated ring‐opening metathesis polymerization. The source of mobility in the solid state is attributed to the plastic crystal nature of dicyclopentadiene, which yields local short‐range mobility due to orientational degrees of freedom. Polymerization in the solid state enables photopatterning, volumetric additive manufacturing of free‐standing structures, and fabrication with embedded components. Solid‐state photopolymerization of dicyclopentadiene offers a new paradigm for advanced and freeform fabrication of high‐performance thermosets.

## Graphical abstract

The solid‐state photopolymerization of dicyclopentadiene is achieved through photoinitiated ring opening metathesis polymerization (ROMP). The kinetics of photopolymerization remain appreciable in the solid state, enabling fabrication methods such as lithography, embedded printing, and volumetric additive manufacturing to be accomplished with solid dicyclopentadiene.

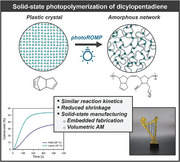

## 1 Introduction

The vast majority of polymerizations are conducted in a liquid, either with neat monomers or with monomers dissolved in a solvent, due to the ease of handling and rapid diffusion of reactants associated with liquid‐state reactions. Polymerization requires mobility of reactive species to ensure bimolecular events such as initiation and propagation can occur at sufficient rates in order to drive the polymerization to high conversion and/or high molecular weights. Diffusion in glassy or ordered crystalline solids is severely restricted, with diffusivities that are orders of magnitude lower than in liquids, making solid‐state polymerization generally infeasible.

However, there are some important counterexamples to this general rule. Topochemical polymerization through cycloaddition reactions of conjugated double bonds, such as cinnamic acids or diacetylene, is contingent on templating of monomers in the crystalline phase, with polymerization converting a monomeric crystalline phase to a polymeric one.^[^ ^1^, ^2^ ^]^ Through alignment of monomeric species in crystal lattices, reactive groups are templated adjacent to one another such that polymerization can proceed with little or no diffusion. Additionally, numerous studies have shown that conventional free‐radical photopolymerization is not only possible in the solid state but can unexpectedly proceed with similar or faster reaction kinetics as compared to the liquid‐state polymerization.^[^ ^3^, ^4^, ^5^ ^]^ In particular, this has been demonstrated in polymerization of (meth)acrylates and other vinylic monomers with crystallizable sidechains, such as long alkyl chains ^[^ ^4^, ^6^, ^7^, ^8^, ^9^ ^]^ or hydrogen bonding moieties.^[^ ^3^ ^]^ He and co‐workers found that vinyl monomers with long alkyl chains such as hexadecyl (C_16_) and octadecyl (C_18_) acrylate form plastic crystal phases,^[^ ^6^, ^7^ ^]^ in this case a rotator phase, in which monomers have rotational degrees of freedom, which enables propagation and polymerization in the solid state. Bowman and co‐workers demonstrated that a cyclic acetal‐functionalized urethane acrylate monomer could be polymerized with faster kinetics in the solid state, which was attributed to the templating of reactive double bonds in the crystalline lattice for facile propagation during polymerization.^[^ ^3^ ^]^ These recent works have also highlighted the significant advantages of photopolymerization in the solid state: reduced shrinkage during polymerization ^[^ ^8^, ^9^ ^]^ and access to higher molecular weights and significantly lower dispersities.^[^ ^4^ ^]^ However, solid‐state polymerization has yet to be utilized as a broader manufacturing or fabrication technique. We posit that the ability to polymerize in the solid state opens up a wide range of possibilities in fabrication and manufacturing. The key enabling feature is the ability to move away from the thin film layer‐by‐layer approach required when processing liquid photocurable systems. This opens up new avenues in polymerization and printing of volumetric elements (also known as volumetric additive manufacturing),^[^ ^10^, ^11^ ^]^ which enables rapid printing. This in stark contrast to the much slower approach of traditional 3D printing, such as vat photopolymerization, in which a 2D thin film is photopolymerized and the 3D object is built up layer‐by‐layer. The use of photocurable precursors in the solid‐state could provide new pathways for mold‐free fabrication of free‐standing objects, volumetric printing, and other compelling fabrication applications.

While research on photopolymerization in the solid‐state has focused exclusively on radical‐mediated processes, there has been great progress in recent years developing alternative chemistries for photopolymerization. Of particular interest is photoinitiated ring‐opening metathesis polymerization (photoROMP), which enables polymerization of a broad palette of monomers into well‐controlled and well‐defined polymers. Significant recent advancements have been made in latent and light‐responsive catalytic systems for photoROMP,^[^ ^12^, ^13^ ^]^ which have been implemented in various applications such as lithography ^[^ ^14^, ^15^, ^16^ ^]^ and additive manufacturing.^[^ ^17^, ^18^, ^19^ ^]^ A common monomer used in these studies is dicyclopentadiene (DCPD), a tricyclic molecule with highly‐strained norbornene and cyclopentene rings, which forms a tough thermoset when polymerized via ROMP. DCPD is a soft, waxy crystalline solid, with commercially‐produced DCPD consisting of >95% of the *endo* isomer.^[^ ^20^ ^]^ This stereoisomer has a melting point of 33 °C and is commonly polymerized with small amounts of a co‐monomer, such as 5‐ethylidene‐2‐norbornene, to depress its melting point and yield a low‐viscosity liquid resin.^[^ ^21^ ^]^ Furthermore, neat DCPD exists in a plastic crystal phase at ambient conditions,^[^ ^22^ ^]^ which suggests the possibility of solid‐state photopolymerization via photoROMP.

**Figure 1:**
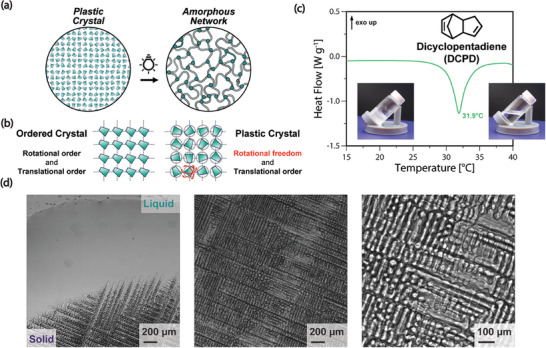
a) Photopolymerization of DCPD in the solid state converts a plastic crystal into an amorphous crosslinked network. b) Plastic crystals are a distinct crystalline phase with plasticity due to weak intermolecular forces which allow for molecular rotational degrees of freedom, in contrast to ordered organic crystalline phases, with both translational and rotational order, leading to brittle crystal phases. c) Heating ramp of DCPD reveals a melting point near ambient of 32 °C, with a transition from waxy solid to low viscosity liquid demonstrated in the inset images. d) Optical micrographs showing recrystallization of DCPD from liquid state at ambient conditions. DCPD plastic crystalline phases demonstrate long‐range order, and pack in a regular cellular fashion.

Taking inspiration from the previous work on solid‐state free radical photopolymerization of vinylic polymers in plastic crystal phases, we hypothesize that the plastic crystalline phases of DCPD might be amenable to photoROMP in the solid state (**Figure** **1**a). We demonstrate from photo‐differential scanning calorimetry (photoDSC) and photorheology data that photopolymerization is indeed possible in the solid‐state for DCPD. Polymerization in the solid‐state enables compelling new manufacturing modalities, such as overmolding of embedded parts not possible via traditional liquid‐state photopolymerization and single‐exposure volumetric printing via greyscale lithography.

## 2 Results and Discussion

We investigated the plastic crystal nature of DCPD and its amenability to solid‐state polymerization via DSC measurements. Plastic crystalline materials are typically composed of globular molecules with long‐range order, but contrast from traditional ordered organic crystalline phases due to significant orientational mobility (Figure 1b). Plastic crystals possess low entropies of melting (*ΔS_m_* < 20 J mol^−1^ K^−1^) and a solid‐solid thermal transition from a lower symmetry crystalline phases to plastic crystal phases at temperatures much lower than the final solid‐liquid transition.^[^ ^23^ ^]^ The grade of DCPD used in this study had a melting point of 32 °C (Figure 1c), transitioning from an opaque waxy solid to a low‐viscosity liquid. Other grades of DCPD were explored (see Figure S1, Supporting Information for melting endotherms), but the Sigma‐Aldrich grade was found to possess the sharpest melting point and was utilized moving forward. The stereoisomer content of this grade of DCPD was confirmed via NMR, with the sample containing 98.4% of the higher melting temperature *endo* isomer (Figure S2, Supporting Information). The *ΔS_m_* (taken as *ΔH_m_/T_m_*) determined from DSC measurements for DCPD was measured to be 7.2 J mol^−1^ K^−1^ and a solid‐solid transition was observed at −56 °C (Figure S1, Supporting Information), further confirming DCPD's plastic crystal nature. X‐Ray diffraction measurements confirmed long‐range ordered crystalline structure, with sharp Bragg‐scattering peaks observed in neat DCPD (Figure S3, Supporting Information). This was further affirmed by optical micrographs of DCPD recrystallizing from above its melting temperature (Figure 1d), forming readily visible microscale crystalline domains, with long‐range order.

**Figure 2:**
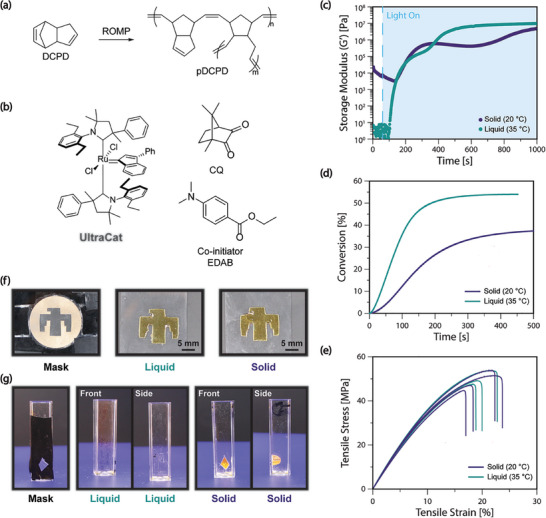
a) Reaction scheme for ROMP of DCPD using b) ruthenium catalyst (**UltraCat**) as photoROMP catalyst, with camphorquinone (CQ) and ethyl 4‐dimethylaminobenzoate (EDAB) utilized as photosensitizer and co‐initiator. c) Photorheology of DCPD resins containing 10000:1 mol. ratio of DCPD:**UltraCat** with blue light (*λ* = 475 nm, light intensity = 20 mW cm^−2^) in liquid and solid state. d) Conversion determined from photoDSC experiments in liquid and solid states with blue light (*λ* = 475 nm, light intensity = 20 mW cm^−2^). e) Stress‐strain curves from uniaxial tensile experiments show similar mechanical properties of photopolymerized samples after thermal post‐cure. f) Lithography using a mask enables reproduction of mask patterns in thin film geometry for both liquid and solid‐state photopolymerization. g) In bulk samples, however, lithography only generates solid crosslinked polymer when DCPD is polymerized in the solid state.

To interrogate the ability of DCPD to be photopolymerized in the solid‐state via photoROMP (**Figure** **2**a), we utilized a previously studied photolatent catalyst and photosensitizer system,^[^ ^19^ ^]^ shown in Figure 2b, that results in rapid photopolymerization and reasonable pot‐life. The system consists of **UltraCat** as the photoROMP catalyst and camphorquinone (CQ) and ethyl‐4‐dimethylamino benzoate (EDAB), as photosensitizer and co‐initiator, respectively. After addition of the catalyst, photosensitizer, and co‐initiator, a slight melting point depression of DCPD (melting point reduced to 30 °C) was observed (Figure S5a, Supporting Information), however the melting point remained above room temperature. Utilizing parallel plate oscillatory photorheology with in situ continuous irradiation (20 mW cm^−2^, 475 nm), the kinetics of photoROMP of DCPD were probed in both liquid (35 °C) and solid (20 °C) states by monitoring the storage modulus over time (Figure 2c). In the liquid state, the DCPD resin prior to irradiation has a storage modulus (G′) below the noise floor of the instrument (< 10 Pa). After irradiation there is a short induction period; continued irradiation results in a rapid rise in storage modulus and gelation (determined in this work by the crossover point between the storage and loss modulus) at 175 s of irradiation time, followed by vitrification of the glassy pDCPD network after ≈400–500 s. In contrast, DCPD in the solid‐state has a comparatively large storage modulus, 10–100 kPa. Upon the first application of the oscillatory shear, a decline in the storage modulus is observed, likely due to localized melting of DCPD at the plate interfaces due to the applied shear stress. However, the storage modulus of solid samples remains orders of magnitude higher than the liquid resins, revealing that the bulk sample still remains in the plastic crystal phase. After irradiation begins, there is a similar induction period before a rapid increase in the storage modulus with polymerization, followed by more gradual increase before eventually reaching the final plateau modulus after 1000 s of irradiation. Polymerization was investigated via photorheology at other temperatures to see the temperature dependence of polymerization in both liquid and solid states (Figure S4, Supporting Information), demonstrating some reduction in reaction rates with the reduction in bulk temperature, but with appreciable photopolymerization occurring down to 10 °C. Additionally, softening of solid‐state DCPD upon application of shear stress was not significant at these reduced temperatures, far away from the melting point. These experiments demonstrate that photopolymerization of DCPD is possible in both liquid and solid states, with only a factor of two difference in the time to reach the plateau storage modulus in the solid state at 20 °C versus polymerizing the liquid resin at 35 °C.

Using photoDSC measurements, the conversion of the DCPD photoROMP reaction was investigated. By using the thermal polymerization of DCPD to determine the overall enthalpy of polymerization (352 J g^−1^, Figure S5, Supporting Information), in agreement with previously reported values for DCPD,^[^ ^24^ ^]^ enthalpy measured isothermally over time during in situ photocuring could be normalized to total enthalpy of polymerization to calculate percent conversion of DCPD. As anticipated, neat DCPD as a control without catalyst or photosensitizer, showed no evidence of polymerization or melting during light exposure (Figure S5b, Supporting Information). With the catalyst and sensitizer, DCPD in solid and liquid states was polymerized, with both samples reaching a plateau in conversion within 500 s of irradiation. DCPD polymerized in the liquid state reached higher conversion (*p_final_* ≈55% for the liquid state vs ≈40% for the solid‐state) and exhibited faster kinetics than the solid state, but both exhibited conversions well above the gel point for DCPD, which occurs at low conversions (*p_c_* ≈1%).^[^ ^25^ ^]^ The influence of catalyst loading was also investigated via photoDSC and photorheology (Figure S5, Supporting Information); lower catalyst loadings resulted in slower kinetics, but still reaching a similar final conversion (*p_final_* ≈35%). The varying final conversions at different polymerization temperatures are likely caused by vitrification of the network, which limits further polymerization. Further reduction in the temperature of solid‐state photopolymerization to 0, −15, and −30 °C resulted in reductions in the final DCPD conversion achievable before vitrification (Figure S5d, Supporting Information), but still demonstrates that significant polymerization is occurring. The temperature during these experiments was also monitored to ensure light exposure and heat of polymerization did not lead to melting (Figure S5h, Supporting Information), with very little deviation of measured temperatures from their setpoints, confirming polymerization in the solid‐state. For bulk samples photopolymerized at room temperature without the temperature regulation provided by the rheology or DSC experimental setups, it is not possible to rule out local melting of the DCPD occurring in polymerized regions from heating originating from the light or the polymerization exotherm.

Photopolymerized samples were post‐cured in an oven in air at 180 °C for 2 h to drive the reaction to full conversion. These post‐cured samples were tested via dynamic mechanical analysis, which showed no difference in crosslink density (with similar rubbery plateau moduli) or glass transition between samples polymerized as liquids and samples polymerized in the solid state (Figure S6, Supporting Information). Uniaxial tensile tests were also performed after post‐cure for photopolymerized samples (Figure 2e), with both types of samples exhibiting similar modulus and strain at break. SEM images of fractured cross‐sections of the post‐cured samples polymerized in solid and liquid states can be seen in Figure S7 (Supporting Information).

Since previous studies had noted that shrinkage was reduced when polymerization occurs in the solid state, the linear (or dimensional) shrinkage during polymerization was measured during photorheology experiments and via dimensional measurements after subsequent post‐cure for liquid and solid DCPD photoresins. We note that this methodology for characterizing polymerization‐induced shrinkage can only assess post‐gelation shrinkage, but due to the very low conversion at the gel point for DCPD, should be reflective of the total linear shrinkage the resin undergoes. In the liquid state, linear shrinkage was 6.5% during photopolymerization, with an additional 0.6% occurring during the post‐cure thermal treatment. In the solid state, 1.7% linear shrinkage was observed during photopolymerization, and an additional 1.5% linear shrinkage occurring in the thermal post‐cure. Nearly a factor of two reduction in polymerization‐induced linear shrinkage can be achieved with polymerization in the solid state instead of a liquid resin.

With photopolymerization, patterning and lithography are predominant areas of application. We investigated the ability to photopattern images via masked light projection in thin films (Figure 2f) and in bulk (Figure 2g). The solid‐state sample showed excellent spatial fidelity to the mask, similar to that of the liquid control in the thin film geometry. However, when photopolymerization was performed on bulk samples in cuvettes, exposure to light in liquid DCPD resins failed to generate a solid part, whereas in solid DCPD resins, photopolymerization generated a crosslinked solid. We attribute this difference to large diffusivity differences between the low‐viscosity liquid resins at 35 °C and the solid‐state resins at 20 °C. In the liquid state, growing polymer chains can rapidly diffuse out of the irradiated region, preventing the local accumulation of polymer chains necessary to grow the network in the defined region to yield a solid part. In the solid state, long‐range diffusion and mobility of polymer species is not possible, but local diffusivity is still high enough to allow for polymerization, enabling a solid part to be photopolymerized in the irradiated region.

**Figure 3:**
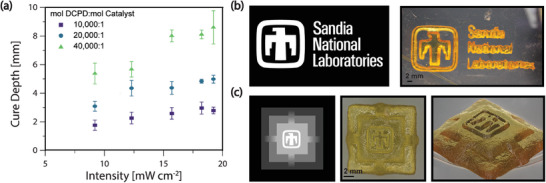
a) Cure depth as a function of light intensity for DCPD with different loadings of **UltraCat** catalyst. All samples were irradiated for 20 min. Error bars are standard deviation, with *n* = 3. b) Greyscale image and generated object from DCPD photopolymerized in the solid state. c) Greyscale image of temple with differing intensity leading to 3D objects with varying topography.

To further explore the ability of solid‐state photopolymerization to fabricate intricate structures, maskless greyscale patterning via a blue‐light (475 nm) digital light processing (DLP) projector was conducted. We probed the cure depth for crosslinked pDCPD photopolymerized in the solid state as a function of light dose at varying **UltraCat** loadings. The catalyst was shown to be the most significant light absorber (Figure S8, Supporting Information), and reductions in loading led to deeper curing. Using light intensity (or total light dose) to control the cure depth, greyscale pattering at different light intensities provided the ability to make tall structures (cure depth up to ≈9 mm) with varying topologies, such as binary patterning for lithography (**Figure** **3**b) or greyscale patterning to create 3D structures (Figure 3c).

**Figure 4:**
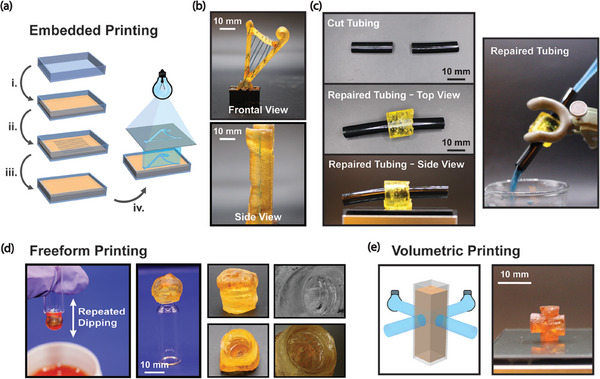
a) Processing protocol for embedding wires in DCPD solid resin and subsequent photopatterned curing: i. initial layer of DCPD is poured and solidified; ii. wires are placed on top of the solidified DCPD resin; iii. Remaining DCPD resin is poured in to fill the mold and solidified; and iv. light projection of harp pattern to photopolymerize object. b) Embedded printing can generate objects with embedded solid elements, akin to the overmolding process used in injection molding. c) The solid‐state nature of the DCPD monomer allows for polymerization while also providing structural integrity, with tubing repaired by conformal recrystallization and photopolymerization of DCDP made water‐tight, allowing flow of dyed water through without leaks (see MV2). d) Once solidified, DCPD can be polymerized in a free‐form manner, generating a conformal cap to a vial. Here, solid resin was applied to the cap by repeated dipping and removing of the vial into a liquid solution of the resin. e) Volumetric printing is possible in a bulk solid sample with light sources at different angles producing different features.

We also explored several applications which had not previously been possible with the use of liquid photoresins. First, we investigated an embedded printing methodology (**Figure** **4**a), in which solid materials were encapsulated within the solidified DCPD resin and then selectively photopolymerized. This was exemplified through printing of a harp with embedded metal wires. A square mold was filled halfway with a layer of melted DCPD resin which was then cooled and solidified. Metal wires were placed on top and were fully supported by the layer of recrystallized DCPD. Then, additional melted DCPD was poured to fill the mold and allowed to solidify at room temperature. Subsequently projecting the harp frame pattern through the entire volume, the resin was selectively polymerized around the embedded wires (Figure 4b). With liquid resins, similar embedded structures would require solid supports for the embedded objects or the addition of rheological modifiers to generate a sufficient yield stress to support the embedded objects during printing. This method can also be applied as a solid‐state photocurable adhesive that possesses an appreciable stiffness and solidity before curing, which allows for sample handling prior to photopolymerization. This approach was demonstrated to repair a cut tube, joining the two cut pieces, and creating a water‐tight repair (Figure 4c, MV2).

Further demonstrations of the utility of solid‐state photopolymerization included freeform manufacturing through mold‐free photopolymerization, which include overhangs and free‐spanning structures. This is exemplified in the fabrication process shown in Figure 4d, in which DCPD resin is applied to a glass vial via multiple iterations of dip coating and solidification and the conformally molded sample is subsequently photopolymerized to produce a cap with threads corresponding to that of the vial (Figure 4d). We do note that some sublimation of DCPD does occur during these longer solid‐state photocuring steps, as DCPD has an appreciable vapor pressure (≈180 Pa at 20 °C) ^[^ ^26^ ^]^ even in the solid state. With the ability to cure at depths up to 9 mm in the bulk solid‐state DCPD resin, we also investigated if solid DCPD could be used for volumetric photopolymerization. Volumetric printing enables rapid printing with a single continuous light exposure ^[^ ^10^, ^11^, ^27^, ^28^ ^]^ and has the potential to drastically reduce fabrication times. We accomplish a volumetric‐type printing via projection from orthogonal angles into a cuvette containing solid DCPD. Printing with a solid resin has the unique benefits of preventing object settling and significantly reducing diffusion of reactive species outside of the irradiated regions. By projecting light from two angles, we demonstrate single‐shot printing of a simple geometry. Further addition of patterning via the use of a projector and inhibition chemistries ^[^ ^18^ ^]^ would allow for far more complex features and resolution.

## 3 Conclusion

For the first time, solid‐state ROMP was demonstrated, with dicyclopentadiene (DCPD) used as the monomeric species. The capacity for polymerization in the solid state was ascribed to the mobility of the rotationally disordered plastic crystalline phase of DCPD. While the kinetics of DCPD photopolymerization were slower in the solid state as compared to liquid state resins, polymerization well past the gel point of DCPD was possible. Solid‐state polymerization yielded high performance thermosets with similar final mechanical properties after thermal post‐curing. Application of solid‐state polymerization of DCPD was illustrated with lithography and volumetric printing. Moreover, freeform photocuring and fabrication with wholly embedded objects – two techniques unachievable with traditional photocuring liquid resins – was demonstrated. The ability to photopolymerize in the solid state will enable new paradigms for support‐free and mold‐free manufacturing with light, with applications spanning advanced manufacturing, embedded electronics, and structural devices.

## 4 Experimental Section

### Materials

Different grades of dicyclopentadiene (DCPD, CAS: 77‐73‐6) were purchased from three different vendors: Oakwood (≥94%), Sigma‐Aldrich (≥96%), and Cymetech (Ultrene‐99, ≥99%). The DCPD from Sigma‐Aldrich was used exclusively unless explicitly stated. Ethyl 4‐dimethylaminobenzoate (EDAB, 99%, CAS: 10287‐53‐3) and was purchased from Oakwood Chemicals. **UltraCat** (CAS: 2055540‐61‐7) and camphorquinone (CQ, 97%, CAS: 10373‐78‐1) were purchased from Sigma‐Aldrich. All chemicals were used as received.

### Representative DCPD Formulation

DCPD was first heated to 50 °C to ensure melting of all crystallites. The ruthenium metathesis catalyst, **UltraCat,** was added, at a 0.01 mol. % loading versus DCPD, to an empty vial. Lower catalyst loadings (0.005 and 0.0025 mol.%) were used in lithography experiments to increase penetration depth. The photosensitizer (CQ) and co‐initiator (EDAB) were added in 5 and 10 wt. equivalents as compared to catalyst. These solids were mechanically homogenized into a fine powder in the vial prior to addition of monomer. DCPD was added using a warmed syringe while constantly stirring the vial to prevent premature polymerization due to high local catalyst concentrations prior to mixing. The solution was then vortexed until solids were completely dissolved. Resins were dispensed as needed in the liquid state and allowed to crystallize prior to use. When not in use, resins were placed in a −20 °C freezer to solidify and extend pot‐life. Pot‐life at ambient conditions was ≈1 h, which was extended to 10 h in the freezer.

### Characterization

*Photorheology*: Rheology experiments were conducted on an ARES‐G2 rheometer (TA Instruments). Photorheology experiments were executed using the TA UV accessory and Thorlabs CHROLIS 6‐Wavelength High‐Power LED Source as the light source. All light intensities were measured using a radiometer (Thorlabs, PM100D with S401C thermal power sensor). The experimental setup consisted of 20 mm diameter parallel plates, with a transparent acrylic top plate and an aluminum bottom plate, with a 0.3 mm gap height. Oscillatory time sweeps were conducted with a 0.5% strain amplitude and a frequency of 1 Hz. Samples were irradiated under continuous light (λ = 365 or 475 nm, *I_0_ = *20 mW cm^−2^) starting 60 s after experiment data collection was started. Sample temperature was maintained using an advanced Peltier system. During experimental runs, a constant axial force of 0 N was maintained and the gap between plates allowed to vary to measure linear shrinkage during photopolymerization, which was defined by the following equation:

(1) $$Linear\;shrinkage\;=\frac{Gap\left(t\;=\;0\right)-Gap\left(final\right)}{Gap\left(t=0\right)}$$

*Differential Scanning Calorimetry (DSC)*: Traditional differential scanning calorimetry was conducted with a Q200 DSC (TA Instruments) under nitrogen atmosphere and at a 10 °C min^−1^ heating and cooling rate. The DSC was calibrated with an indium standard.

*Photocalorimeter Differential Scanning Calorimetry (Photo‐DSC)*: Photo‐DSC was conducted on a Q200 DSC (TA Instruments) equipped with a photocalorimeter accessory (TA Instruments part 935 000.901). The DSC was calibrated with an internal indium standard. Photocalorimetry was performed using a Thorlabs CHROLIS 6‐Wavelength High‐Power LED Source with a 475 nm LED. The tests were conducted at 20 mW cm^−2^ as measured by a radiometer in a geometry mimicking the photo‐DSC experimental geometry. Resin samples (≈10 mg)  were placed in an aluminum DSC pan with no lid and tested under the flow of nitrogen (50 mL min^−1^). Isothermal tests were run at the temperatures of interest with continuous irradiation starting one minute after temperature equilibrium was reached and ending upon plateau of the exotherm. Conversion at a given time was calculated by dividing a running integral of the exotherm by the heat of polymerization as found by a thermal cure (352 J g^−1^, Figure S5a, Supporting Information). The running integral was taken from the area above the plateau value to correct for heat capacity.

*Tensile Testing*: Tensile testing was performed using an electromechanical load frame (Instron 5982). Dogbone specimens (ASTM type V) were produced by projecting blue light from a 475 nm DLP projector (EKB Technologies Ltd) at a light intensity of 20 mW cm^−2^ onto DCPD resin in between two glass slides with a 0.6 mm spacer for 5 min. Dogbone specimens were further thermally cured at 180 °C for 2 h and tested at a strain rate of 5% min^−1^.

*Dynamic Mechanical Testing (DMA)*: Variable temperature DMA was performed using a strain‐controlled ARES‐G2 (TA Instruments) rheometer operating in small‐amplitude oscillatory shear mode. The dynamic shear storage (G′) and loss (G′′) moduli and loss tangent (G′′/G′) of thin rectangular plaques formed using a mold were measured under torsional strain with 1 Hz oscillation frequency and 0.5% strain amplitude. Measurements were conducted heating from 50 to 200 °C with a ramp rate of 3 °C min^−1^.

*UV–Visible Spectrophotometry*: UV–visible spectrophotometry was performed using an Agilent Technologies Cary UV–vis spectrophotometer. Spectra were collected from 200 to 800 with 1 nm resolution on solutions using a 10 mm path length quartz cuvette. A cuvette containing DCPD monomer was used as a reference.

*Powder X‐Ray Diffraction (pXRD)*: Powder X‐Ray Diffraction pPXRD) spectra were taken using a Bruker D8 VENTURE diffractometer with Mo Kα radiation (λ  =  0.71070 Å). Samples were taken and placed upon a silicon wafer sample holder. The scanning step was 0.08° in range of 2θ  =  10° to 90°.

*Nuclear Magnetic Resonance Spectroscopy (NMR)*: ^1^H NMR spectroscopy was performed on a Bruker Avance III 500 MHz instrument in CDCl_3_. Percent of *endo* stereoisomer was determined by relative peak area from integration of the CH_2_ peak for *endo‐*DCPD between 5.53 and 5.45 ppm and a CH peak for *exo‐*DCPD between 5.77 and 5.7 ppm, as per prior literature.^[^ ^29^ ^]^

*Cure Depth Experiments*: Thick films of dicyclopentadiene were placed in a mold and capped with a glass slide and then photopolymerized via 475 nm projector light source with varying grayscale values to achieve different intensities with the same light exposure time (20 min) to generate cured films that had been exposed to different light doses. The cure depth was measured with calipers and was reported as the mean of 3 samples, with the error given as one standard deviation from the mean.

### Lithography and Volumetric Printing

Lithography, volumetric printing, and greyscale patterning was conducted using a 475 nm DLP projector (EKB Technologies Ltd) at a light intensity of 20 mW cm^−2^ and exposure times of 20 to 30 min. Parts were post‐cured at 160 °C for 2 h.

## Conflict of Interest

The authors in this article have intellectual property related to the technology described.

## Supporting information

Supporting Information

Supplemental Video 1

Supplemental Video 2

## Data Availability

The data that support the findings of this study are available from the corresponding author upon reasonable request.
