# Correction to “Volumetric Additive Manufacturing of Dicyclopentadiene by Solid‐State Photopolymerization”

**DOI:** 10.1002/advs.75656

**Published:** 2026-05-11

**Authors:** Matthew M. Hausladen, Esteban Baca, Kyle A. Nogales, Leah N. Appelhans, Bryan Kaehr, Craig M. Hamel, Samuel C. Leguizamon

**Affiliations:** ^1^ Chemical Engineering and Materials Science University of Minnesota Minneapolis Minnesota USA; ^2^ Sandia National Laboratories Albuquerque New Mexico USA

Hausladen, Matthew M., et al. “Volumetric Additive Manufacturing of Dicyclopentadiene by Solid‐State Photopolymerization.” *Advanced Science* 11.34 (2024): 2402385.

https://doi.og/10.1002/advs.202402385

In the acknowledgments, a funding source was omitted. The authors acknowledge the US DOE: “This work was supported in part by the Regenerative Energy‐Efficient Manufacturing of Thermoset Polymeric Materials (REMAT), an Energy Frontier Research Center funded by the US DOE, Office of Science, Basic Energy Sciences at the University of Illinois, Urbana‐ Champaign under award no. DE‐SC0023457 and at Sandia National Laboratories under contract DE‐NA0003525.”

We apologize for this error.

