# Supplementary material for: Volumetric Additive Manufacturing of Dicyclopentadiene by Solid‐State Photopolymerization
Source: Adv Sci (Weinh). 2024 Jul 5;11(34):2402385. doi: 10.1002/advs.202402385 (PMC11425911; doi:10.1002/advs.202402385)
Supplement: Supplementary file 1 — Supporting Information [file ADVS-11-2402385-s002.docx]

Supporting Information for

**Volumetric additive manufacturing of dicyclopentadiene by solid-state photopolymerization**

Matthew M. Hausladen^1^*, Esteban Baca^2^, Kyle A. Nogales^2^, Leah N. Appelhans^2^, Bryan J. Kaehr^2^, Craig M. Hamel^2^, Samuel C. Leguizamon^2^*

^1^University of Minnesota, Chemical Engineering and Materials Science, Minneapolis, Minnesota, 55455, United States

^2^Sandia National Laboratories, Albuquerque, New Mexico 87185, United States

*Corresponding authors

Email: Matthew M. Hausladen – hausl021@umn.edu

Samuel C. Leguizamon – sleguiz@sandia.gov

**
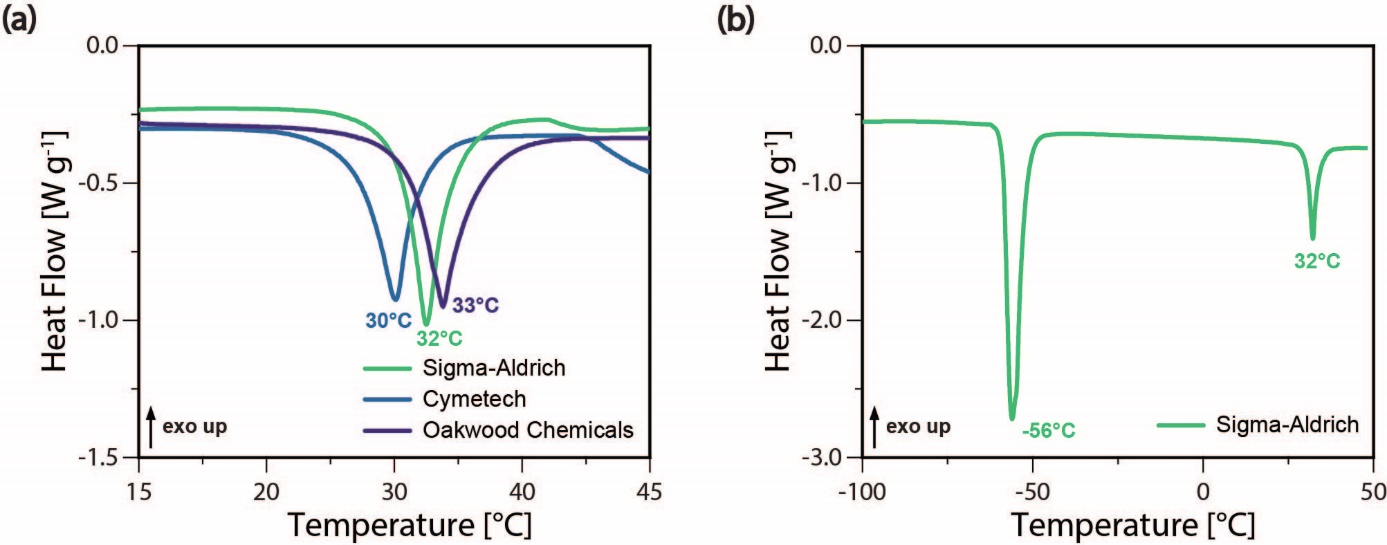
**

**Figure S1. a)** DSC thermograms of heating cycle of different grades of DCPD from three different suppliers, with melting temperatures for each. **b)** Low-temperature DSC scans of Sigma-Aldrich DCPD showing solid-solid phase transition at -56°C.

**
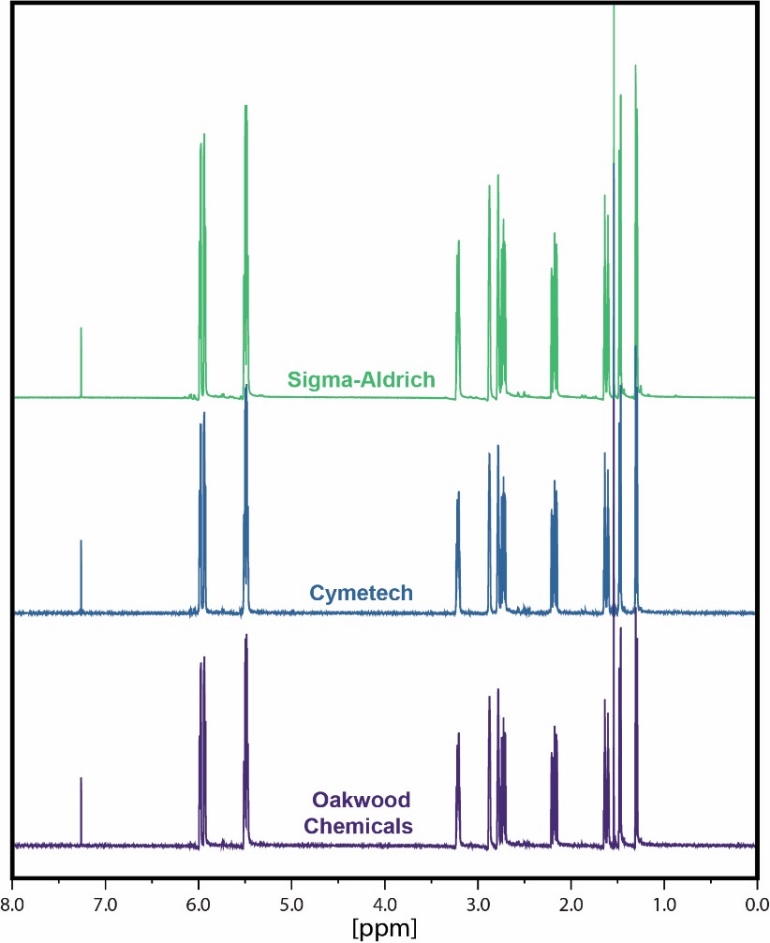
**

**Figure S2.** NMR spectra of different DCPD grades from various suppliers, with the following endo-DCPD contents: Cymetech (97.6% endo), Sigma (98.4%), Oakwood (98.5%). Percent of endo stereoisomer was determined by relative peak area from integration of the CH_2_ peak for endo-DCPD between 5.53 and 5.45 ppm and a CH peak for exo-DCPD between 5.77 and 5.7 ppm, as per prior literature.^[28]^


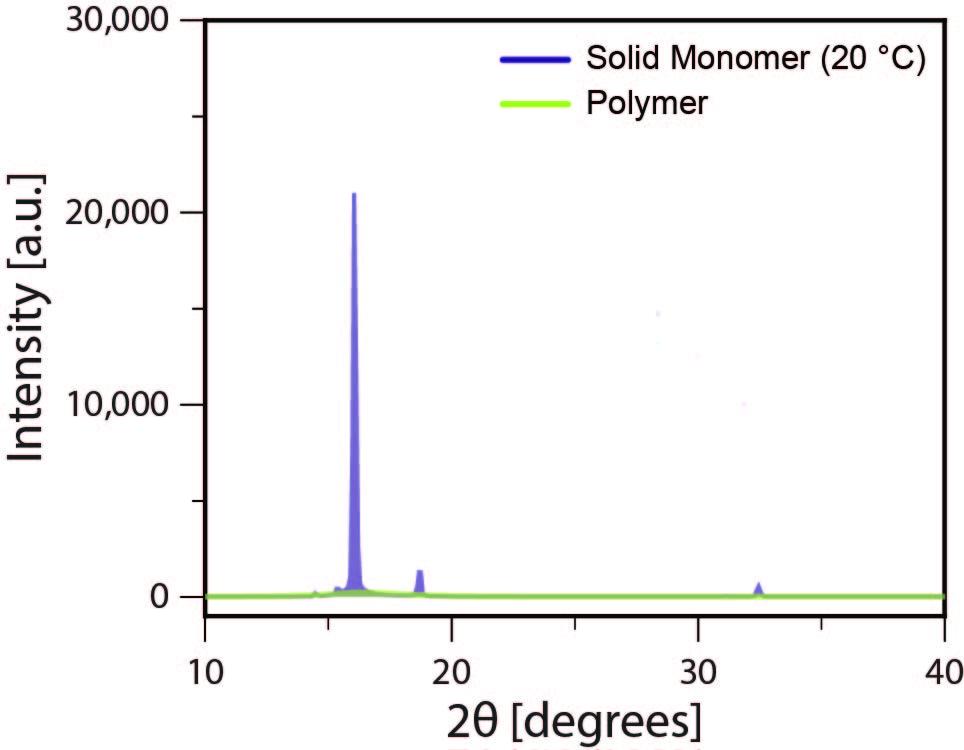


**Figure S3.** X-ray diffraction (XRD) of neat solid DCPD monomer and post-cured, crosslinked poly(DCPD) polymer films. No literature PXRD or single crystal XRD patterns for DCPD were found for comparison.


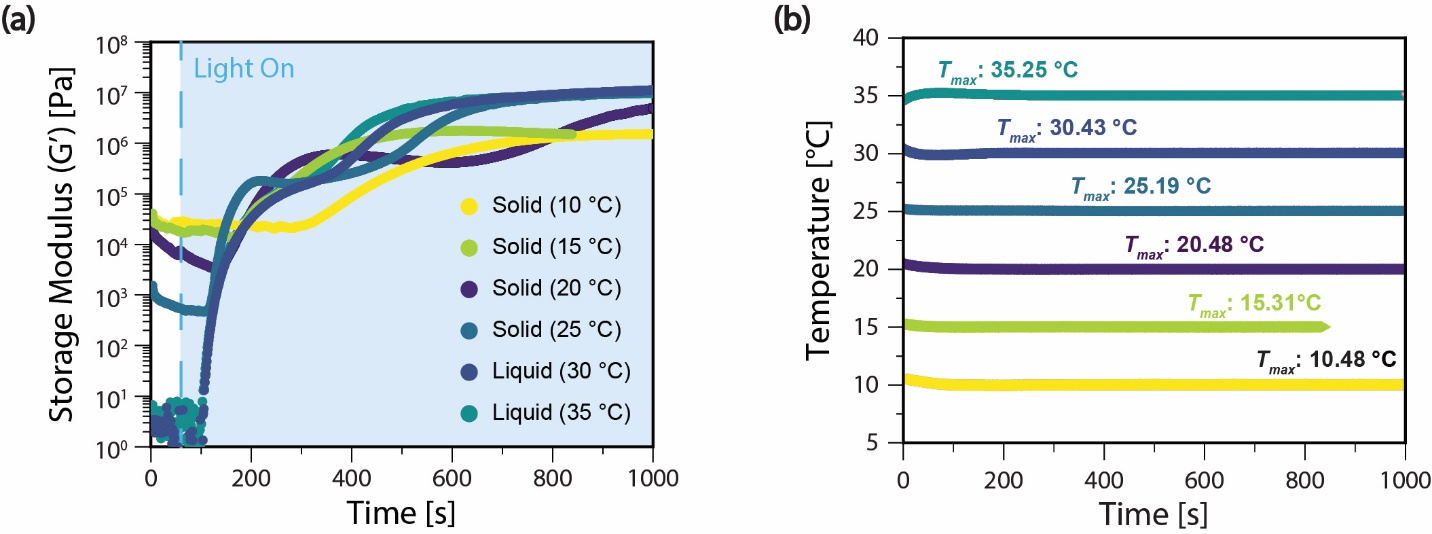


**Figure S4. (a)** Photorheology of DCPD at different temperatures with irradiation (475nm, 20 mW/cm^2^) beginning at 60s. **(b)** Measure temperatures during photorheology experiments show some initial slight fluctuations in temperature due to light and polymerization exotherms, but none exceeded 0.5°C from the set point temperature.

**
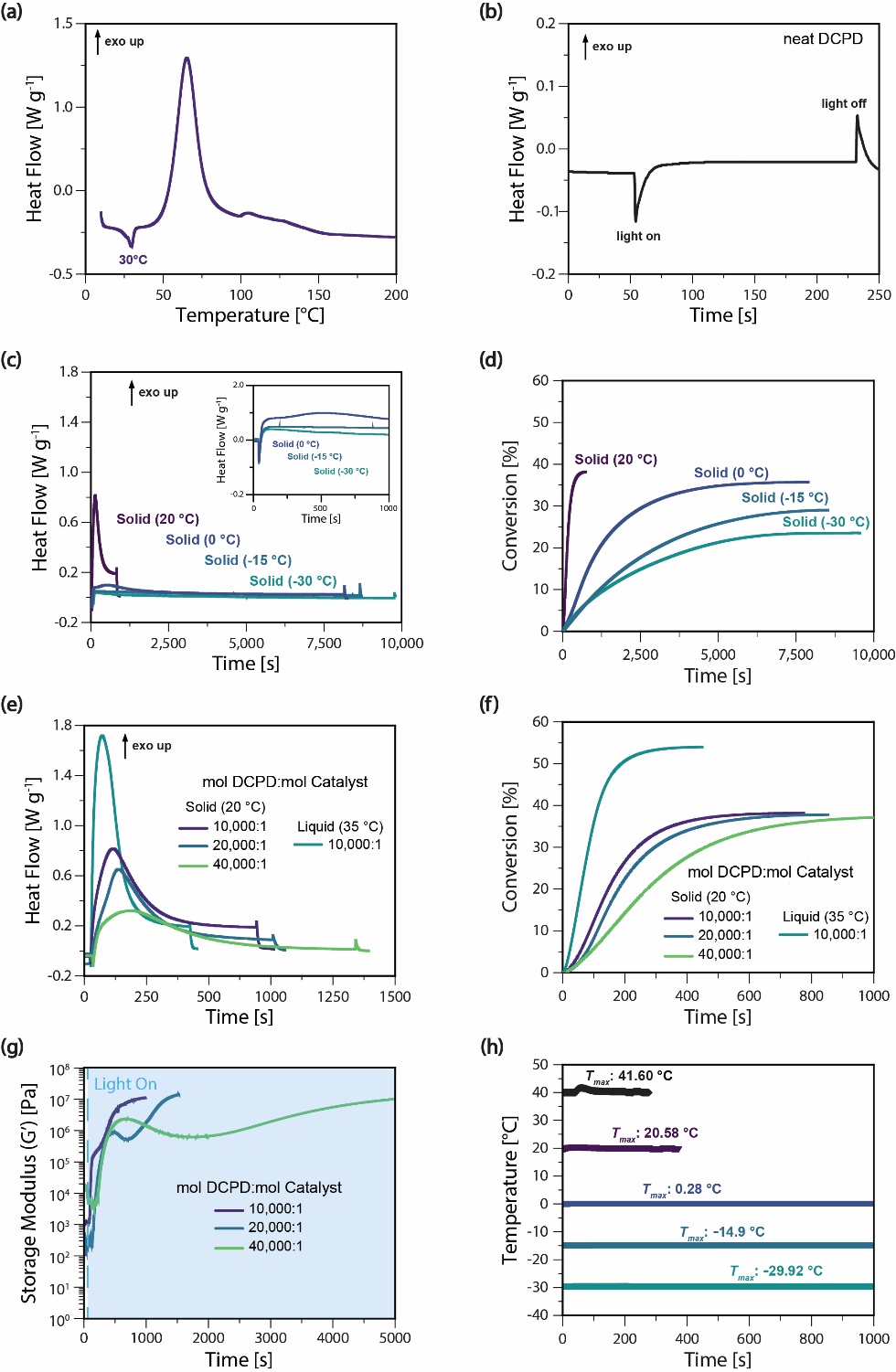
**

**Figure S5. (a)** DSC heating ramp thermogram of DCPD with 0.01 mol.% catalyst, illustrating melting endotherm (*ΔH_m_* = 1.22 kJ/mol) at 30°C, followed by polymerization exotherm (*ΔH_p_* = 46.5 kJ/mol). **(b)** Control run in photoDSC with neat DCPD exposed to light, with no exotherm or endotherm related to melting or polymerization observed. **(c)** PhotoDSC experiments and **(d)** conversion of solid-state samples irradiated with blue light (using 475 nm, 20 mW/cm^2^) at 0 and 20°C. **(e)** PhotoDSC experiments irradiated with blue light (using 475 nm, 20 mW/cm^2^) in liquid state and solid-state (with different catalyst loadings), show different reaction rates when converted to **(f)** conversion. **(g)** Photorheology with different catalyst loadings in the solid-state at 20°C. **(h)** Measured temperatures from photoDSC show very little deviation from setpoint due to light exposure or polymerization exotherm.

**
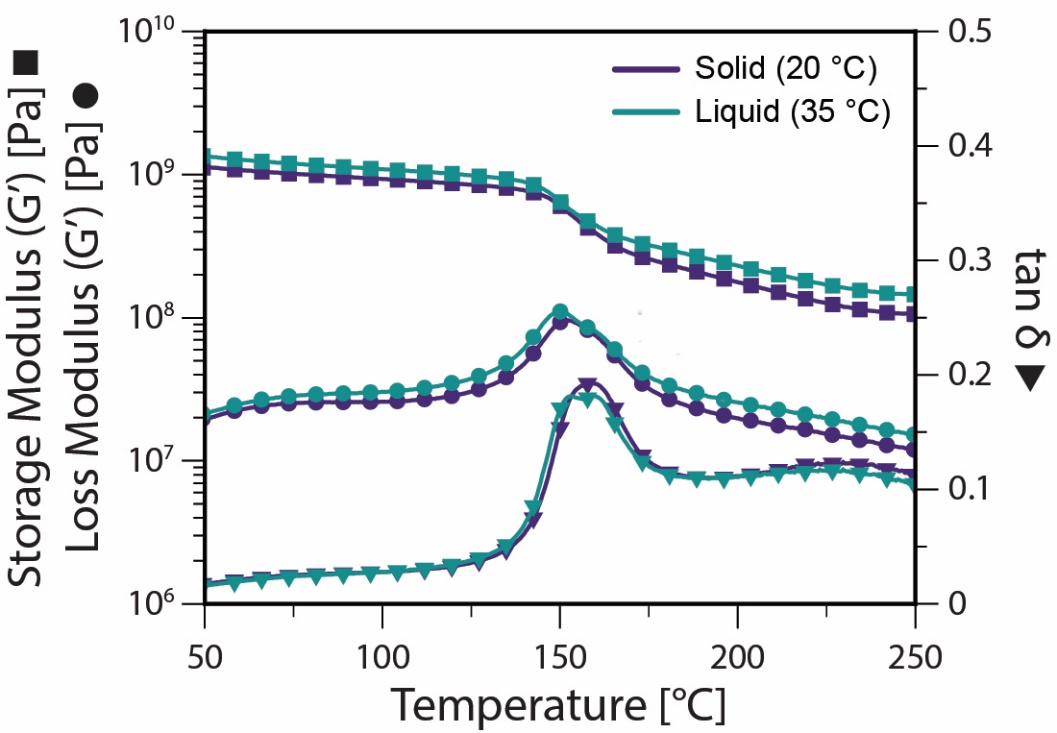
**

**Figure S6.** DMA of thermally post-cured DCPD samples (180 °C, 2 hours) photopolymerized in different states.


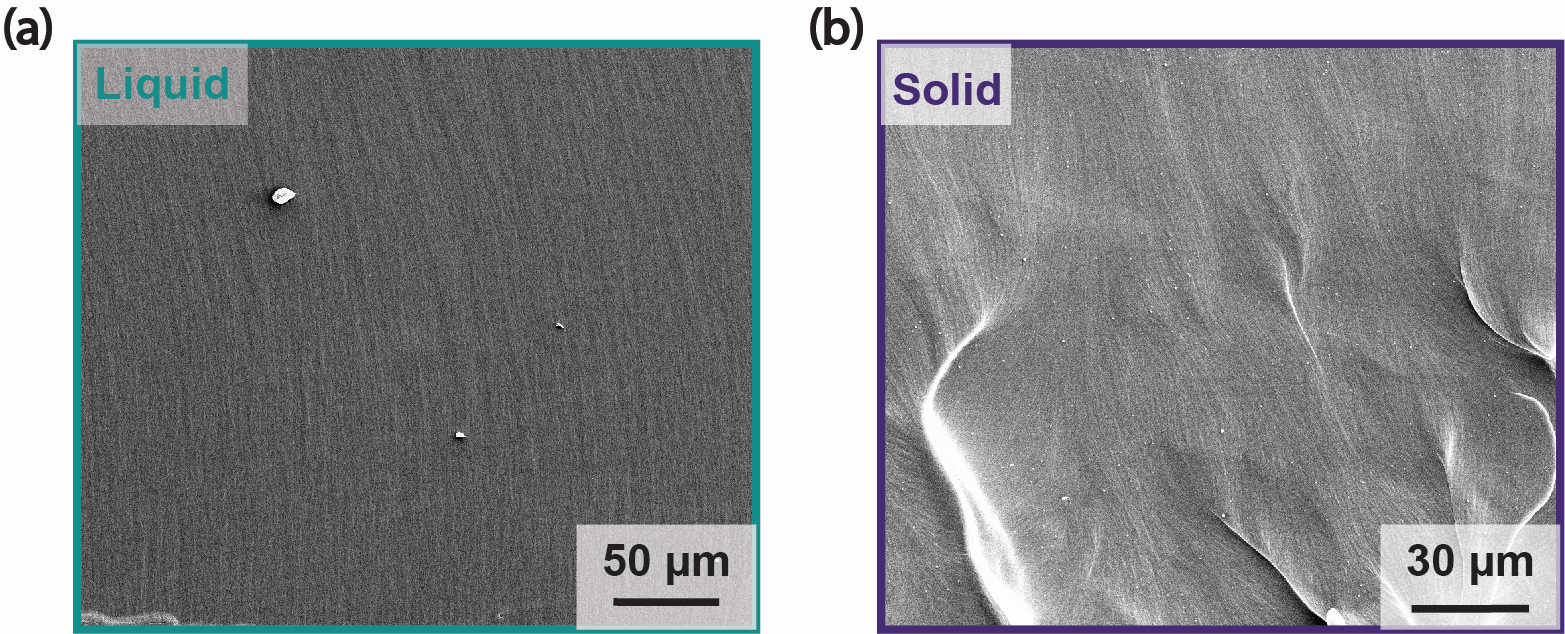


**Figure S7.** SEM images of fracture surfaces from post-cures samples photopolymerized in **(a)** liquid and **(b)** solid-state.


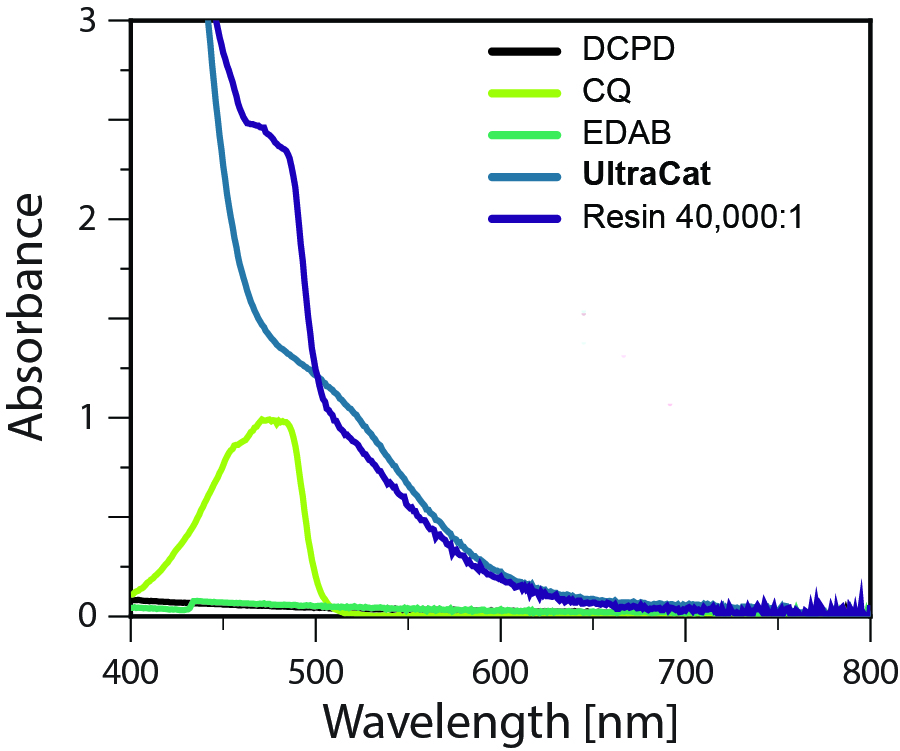


**Figure S8.** UV-VIS of various resin components, with the **UltraCat** catalyst yielding the most significant contributions to light absorption. Spectra were taken at resin equivalent concentrations.
